# A case report and literature review of Carney complex with atrial adenomyxoma

**DOI:** 10.1186/s12902-023-01285-7

**Published:** 2023-02-06

**Authors:** Jing Xu, Meng Ye, Po Li, Shujing Xu, Miao Zhang, Lixin Shi, Juan He

**Affiliations:** 1 Department of Endocrinology and Metabolism, the Affiliated Hospital of Guizhou Medical University, Guiyang, 550004 China; 2 Department of Pathology, the Affiliated Hospital of Guizhou Medical University, Guiyang, 550004 China; 3 Department of Endocrinology and Metabolism, Guiqian International General Hospital, Guiyang, 550004 China

**Keywords:** Carney complex, Adenomyxoma, Cushing syndrome, *PRKAR1A* gene, Case report

## Abstract

**Background:**

Carney complex (CNC) is a rare multiple endocrine neoplasia syndrome characterized by mucocutaneous lentigines/ blue nevi, cardiac myxoma and endocrine overactivity. Here, we report a CNC case with *PRKAR1A* gene mutation characterized by left atrial adenomyxoma to explore the diagnosis and treatment of CNC.

**Case presentation:**

A 42-year-old woman with a history of cardiac tumour surgery presented with typical features of Cushing syndrome, including central obesity, buffalo hump, mild facial plethora, purple striae on the lower abdomen, and spotty skin pigmentation. Left atrial adenomyxoma and thyroid papillary carcinoma were identified by postoperative histologic assays. Genetic screening revealed a pathogenic germline heterozygous mutation of c.682C > T (p.R228X) in exon 7 of the *PRKAR1A* gene. The clinical features and normal ACTH levels suggest this patient suffered the ACTH-independent primary pigmented nodular adrenocortical disease (PPNAD) with cyclic hypercortisolism or ACTH-dependent Cushing syndrome.

**Conclusion:**

CNC is uncommon, however, if a patient develops clinical features involving multiple endocrine and non-endocrine tumors, especially Cushing syndrome and cardiac myxoma, CNC should be considered. Genetic analysis is recommended in patients with suspected CNC.

**Supplementary Information:**

The online version contains supplementary material available at 10.1186/s12902-023-01285-7.

## Background

Carney complex (CNC) is a rare autosomal dominant multiple endocrine neoplasia syndrome characterized by mucocutaneous lentigines/blue nevi, cardiac myxoma, and endocrine overactivity [1]. Patients may present with two or more endocrine tumors, including primary pigmented nodular adrenaocortical disease (PPNAD), growth-hormone-secreting pituitary adenoma or prolactinoma, thyroid adenoma or carcinoma, and gonadal tumors. Non-endocrine tumors associated with CNC include myxomas of the heart, skin or breast [2]. Mutations in the *PRKAR1A* gene encoding the cAMP-dependent protein kinase A (PKA) type 1α subunit have been identified in more than 70% of CNC cases [3]. Here, we report a CNC case with mutated *PRKAR1A* which was characterized by left atrial adenomyxoma.

## Case presentation

A 42-year-old woman was admitted to our department because of elevated blood glucose and blurred vision. This patient was diagnosed with diabetes in a local hospital during pregnancy 7 years ago, then developed blurred vision and extremity numbness. She was treated with metformin and underwent a cardiac mass surgery in our hospital 3 years ago. Postoperative histologic assays indicated this patient may have suffered a “left atrial mucinous adenocarcinoma”. In addition, the patient had a history of hypertension for over 10 years and was treated with enalapril maleate. Her menarche was at the age of 15, having a menstrual period and cycle of 5–7 and 27–30 days, respectively. She had been experiencing menstrual disorders 1 year prior to this admission, manifested as a longer menstrual cycle (2–4 months) and decreased menstrual flow (2–3 days). The patient denied having relatives with a similar medical family history.

**Fig. 1:**
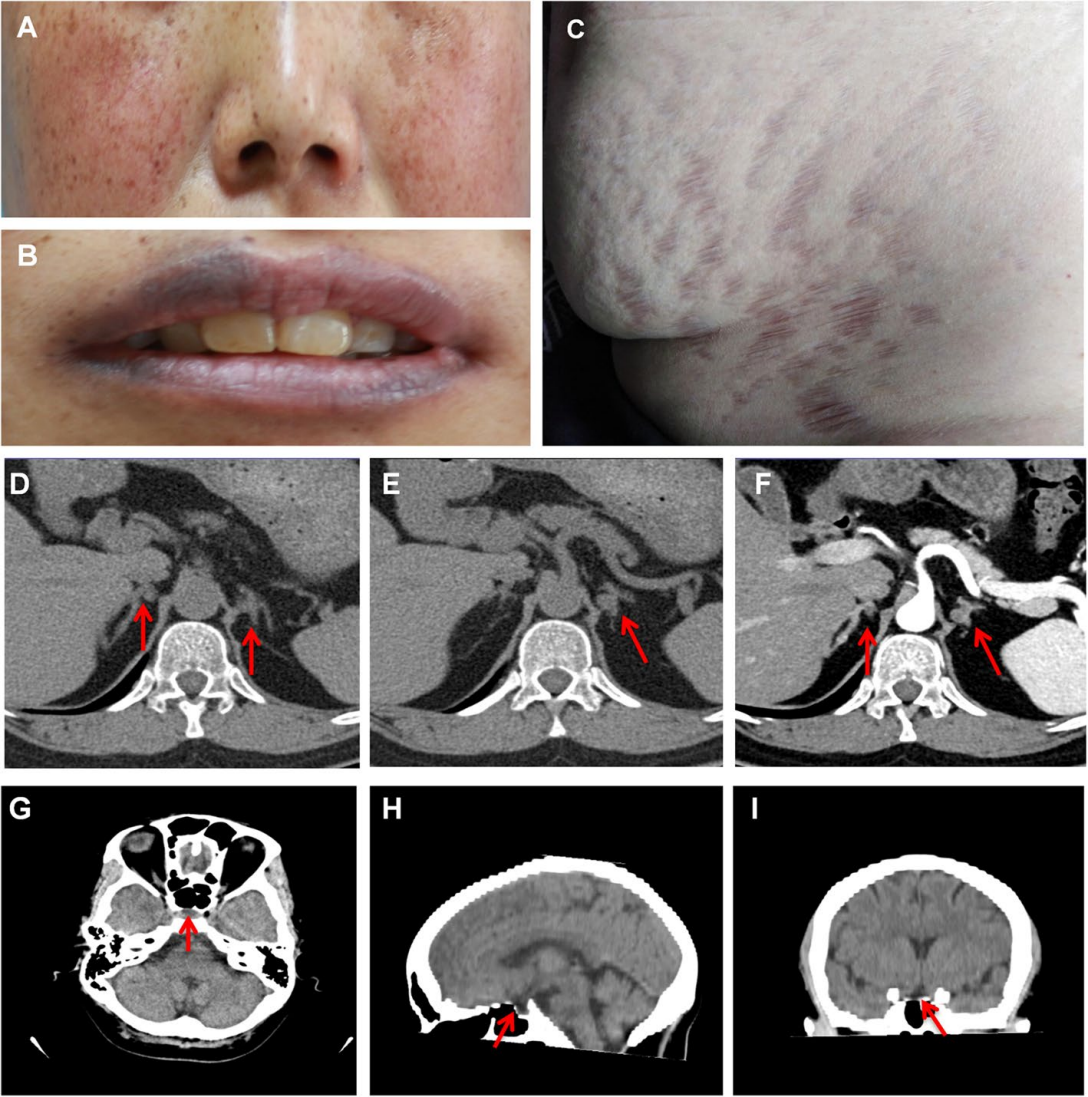
Clinical characteristics and imaging of the patient. (**A**) Mild facial plethora, pigmented spots on the face; (**B**) Pigmented spots on lips; (**C**) Purple striae on the lower abdomen. (**D**-**F**) Adrenal computed tomography (CT) showed multiple small nodules on both sides of the adrenal glands (the larger diameter is 8 mm), considered adenomas; (**G**-**I**) Brain CT showed the ischaemic focus of the left half oval centre and the partial empty sella

**Fig. 2:**
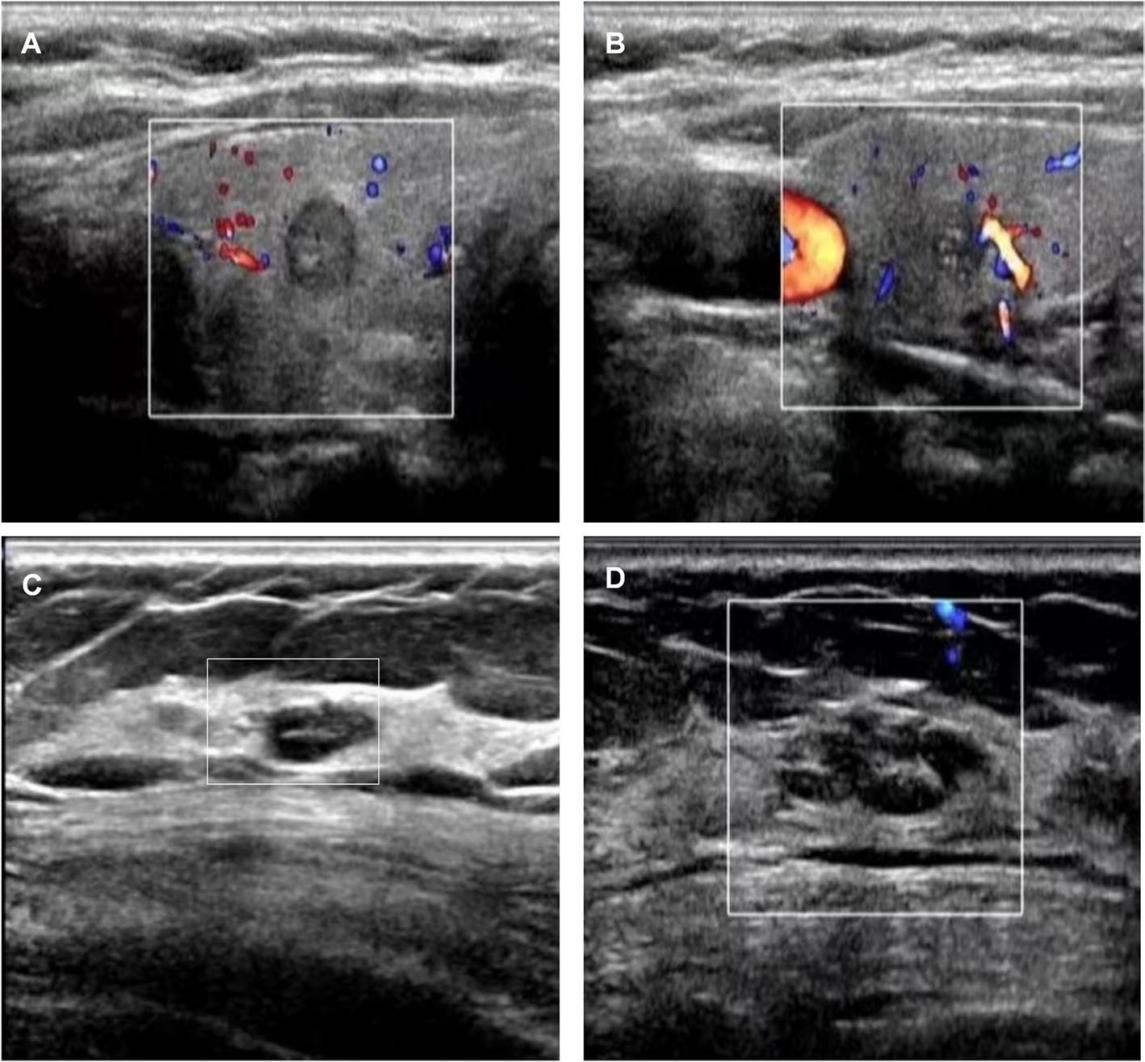
Thyroid ultrasound and breast ultrasound. (**A**) Preoperative thyroid ultrasound revealed a slightly hypoechoic nodule in the right thyroid, 6 × 6 mm in size, regular in shape, clear in boundary, punctate blood flow signals, and microcalcifications, with a thyroid imaging reporting and data system (TI-RADS) classification of 4a. (**B**) Preoperative thyroid ultrasound revealed a hypoechoic nodule in the right thyroid, 8 × 5 mm in size, irregular in shape, less clear in boundary, rich in blood flow signals, and microcalcifications, with a TI-RADS classification of 4b. (**C**) Breast ultrasound at the first visit showed the left breast with multiple hypoechoic nodules, maximum size of 9 × 5 mm, regular in shape, clear in boundary, no blood flow signal, with a breast imaging reporting and data system (BI-RADS) classification of 3. (**D**) Breast ultrasound at the first visit showed multiple hypoechoic nodules in the right breast, one of which was tumor-like hyperplasia, 18 × 7 mm in size, less regular in shape, less clear in boundary, no blood flow signal, with a BI-RADS classification of 4a

A physical examination revealed typical features of Cushing syndrome (centripetal obesity, buffalo hump, mild facial plethora and purple striae on the lower abdomen) and spotty pigmentation of facial skin and lips (Fig. 1 A-C). Laboratory findings are detailed in Table 1. Adrenal and brain computed tomography (CT) scans are shown in Fig. 1 D-F and Fig. 1 G-I, respectively. A cardiac ultrasound revealed tricuspid regurgitation. A thyroid ultrasound revealed a 6 × 6 mm slightly hypoechoic nodule in the right thyroid, classified as 4a by the thyroid imaging reporting and data system (TI-RADS), whilst another 8 × 5 mm hypoechoic nodule in the right thyroid, as TI-RADS classification 4b (Fig. 2 A, B). Thyroid fine needle aspiration (FNA) biopsy was suspicious for right papillary thyroid carcinoma, Bethesda V (Figure S1). A breast ultrasound showed a hypoechoic nodule in the right breast suspicious for tumor-like hyperplasia with a breast imaging reporting and data system (BI-RADS) classification of 4a; double breasts with multiple hypoechoic nodules with a BI-RADS classification of 3 (Fig. 2 C, D)**.** Furthermore, a vaginal ultrasound showed a cystic mass in the left ovarian attachments and cervical Nessler's cyst. The bone mineral density of the lumbar vertebrae (L1, L2, L3, L4) of the patient was measured by dual-energy X-ray absorptiometry (DXA). The Z values (compared with a population of the same race, gender and age group) of the lumbar vertebrae were -3.2, -2.4, -3.1 and -2.4 for L1, L2, L3 and L4, respectively, and the total Z value of the lumbar vertebrae was -2.8, clearly indicating the presence of osteoporosis.

**Table 1:** Laboratory parameters of the patient

Laboratory parameters	First visit	1 year	Reference ranges	Unit
Fasting Glucose	5.64	6.49	3.90–6.10	mmol/L
HbA1c	6.9	7.1	4.3–5.7	%
LH	7.32	33.22	1.90–12.50	IU/L
FSH	44.01	51.31	2.50–10.20	IU/L
Estradiol	114.87	293.80	71.60–529.20	pmol/l
Progesterone	0.58	0.94	0.48–4.45	ng/ml
PRL	450.51	443.00	59.00–619.00	mIU/L
Testosterone	0.63	0.09	0.29–1.67	nmol/L
ACTH 8:00 am	24.40	45.40	5.00–46.00	pg/ml
Cortisol 8:00 am (baseline)	11.21	11.40	4.30–22.40	µg/dl
Cortisol 8:00 am (1 mg dexamethasone suppression)	10.06	-	<1.8	µg/dl
Cortisol 8:00 am (low-dose dexamethasone suppression)	10.02	12.40	<1.8	µg/dl
Cortisol 8:00 am (high-dose dexamethasone suppression)	11.19	13.70	Suppression>50% of baseline	µg/dl
TSH	0.566	2.070	0.270–4.200	mIU/L
FT3	4.10	4.50	3.10–6.80	pmol/L
FT4	16.50	17.93	12.00–22.00	pmol/L
CA125	44.47	44.37	0.00–35.00	U/ml
CA199	29.82	45.30	0.00–27.00	U/ml
CEA	5.07	6.96	0.00–6.50	ng/ml
Thyroglobulin	11.50	9.20	0.73–55.00	ng/ml

**Fig. 3:**
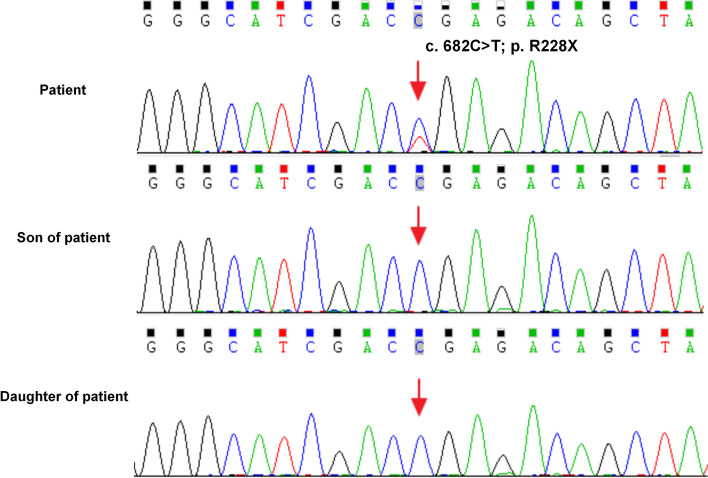
The *PRKAR1A* gene sequence of the patient and her children. Sequencing of DNA extracted from peripheral blood identified a heterozygous mutation (c. 682C > T) in *PRKAR1A* exon 7, which changed the original synthetic arginine codon into a stop codon (p. R228X, red arrow). The patient's children were negative

**Fig. 4:**
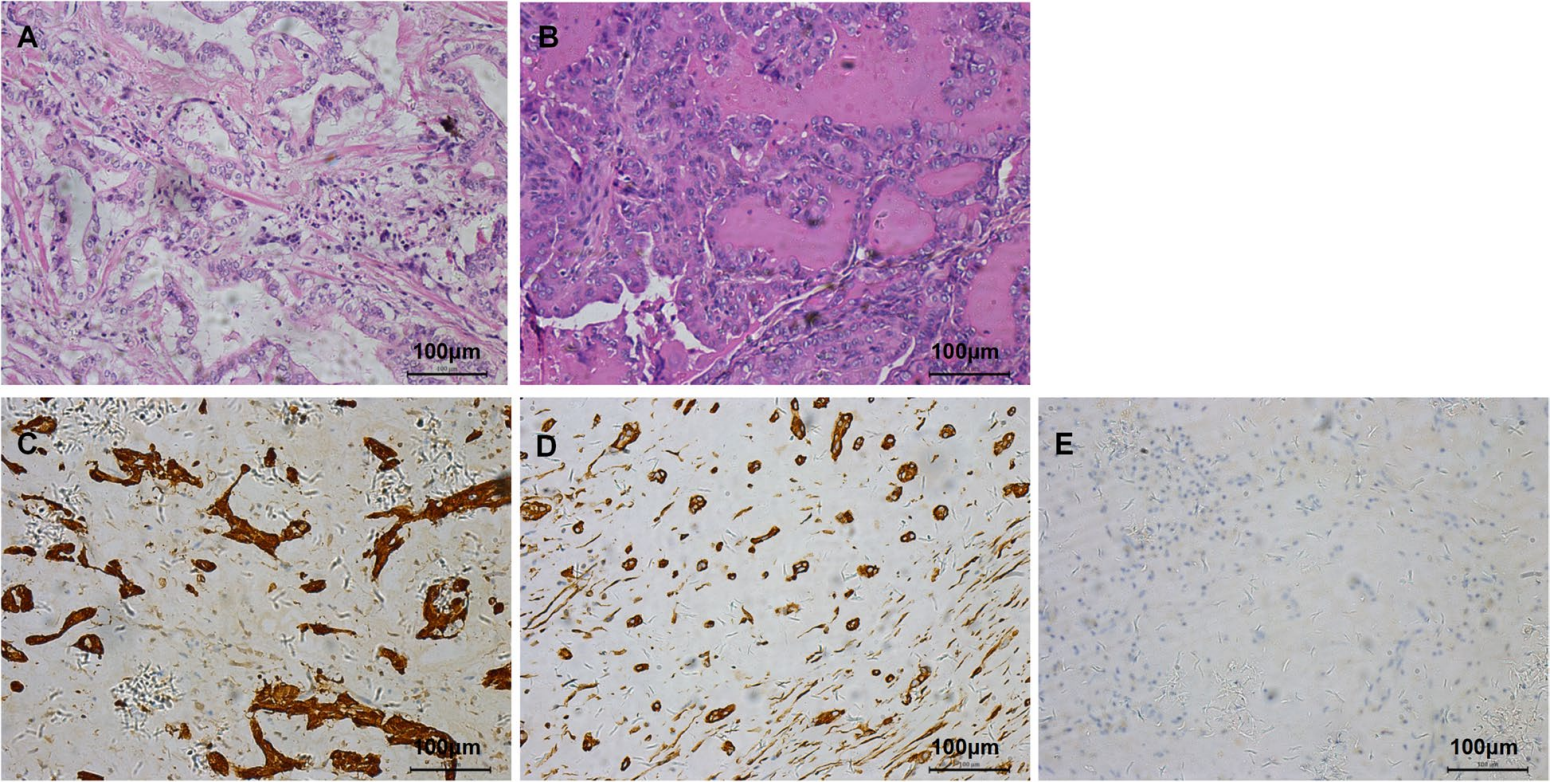
Histopathology profiles. (**A**) Haematoxylin and eosin (HE) staining of the cardiac mass tissue showed atypical gland infiltration in myocardial muscle bundles and small nest or strand-like infiltrating growth in atypical mucus epithelium. (**B**) HE staining of thyroid nodules suggested papillary thyroid carcinoma. Immunohistochemistry of the cardiac mass tissue including strong immune reactivity to calretinin (**C**), vimentin (**D**), and faint reactivity to MOC-31 (**E**)

Consented by the patient, her peripheral blood was collected and sent to the Maikino Medical Laboratory (Beijing, China). Mutation in *PRKAR1A* was detected by the whole exome sequencing and verified by the Sanger sequencing using the forward (5’-TCGTCAGAAATCACCTATTCTTCTC-3’) and reverse (5’-GCTAAGCTGGGCTTAATGCAA-3’) primers. A known pathogenic germline mutation of the *PRKAR1A* gene (c. 682C > T) in exon 7 was found. This point mutation introduced a premature stop codon that substitutes the arginine (Arg) codon (p. R228X) in *PRKAR1A*. Consequently, the mutated *PRKAR1A* can only translate a C-terminus truncated loss-of-function PRKAR1A polypeptide in CNC. However, children (son and daughter) of this patient did not carry this mutation (Fig. 3). The cardiac tumor that occurred 3 years ago was rechecked and finally confirmed as "left atrial adenomyxoma" via Hematoxylin and eosin (HE) staining (Fig. 4 A) and immunohistochemistry (Fig. 4 C, D, E). All the pathology images were taken using Leica DM6B upright microscopes and the Leica Application Suite as the acquisition software. The resolution of each original image is 300dpi and there is no downstream processing or averaging that enhances the resolution of the image.

The patient was then transferred to the surgery department of our hospital for radical thyroid surgery undergoing right thyroid gland lobe removal, isthmus and central lymph node dissection plus right recurrent laryngeal nerve exploration under general anesthesia. The postoperative histopathology indicated papillary carcinoma of the right thyroid (Fig. 4 B). Based on the clinical symptoms, physical examination and relevant laboratory results, the patient’s adrenal lesions were considered to be PPNAD-associated Cushing syndrome and bilateral adrenalectomy was recommended. However, due to personal reasons, she refused any further surgery. Adrenal steroidogenesis inhibitors, including ketoconazole, metyrapone, and osilodrostat, are recommended for medical therapy when surgery is not feasible [4]. However, these drugs are not available in China. Additionally, the glucocorticoid receptor blocker mifepristone can also improve key clinical features associated with hypercortisolism, specifically hyperglycemia and weight gain [4]. However, it is only used as an abortifacient in the first months of pregnancy in China. Therefore, the appropriate treatments were given to protect against Cushing syndrome-related complications, including glycemic control (Metformin 2.0 g/d and Acarbose 150 mg/d), blood pressure control (Enalapril Maleate 10 mg/d, Metoprolol Succinate Sustained-release Tablet 23.75 mg/d), and osteoporosis management (Calcium Acetate 0.6 g/d and Alendronate Sodium/Vitamin D3 combination Tablet, 1 tablet/week). The patient was followed up 1 year later, and some relevant results are shown in Table 1 and Figure S2. She was perimenopausal at that time. Despite a previously suspected malignant nodule in the right mammary gland, a re-examination via ultrasound considered the nodule to be benign (Figure S2 C).

## Discussion and conclusions

In 1985, Carney first described a syndrome characterized by myxoma, skin pigmentation and endocrine overactivity [1]. The incidence of CNC is unclear. A previous study reported more than 750 cases of CNC, including whites, African Americans and Asians from the Americas, Europe and Asia [5]. Another study enrolled 353 patients showed that 63% of cases were women [6]. The progression of CNC takes many years. Patient age at diagnosis ranges from newborn to adults in their 50 s (the median age is 20 years) [5].

The criteria for the diagnosis include major and supplementary criteria, which are listed in Table 2. Patients who meet any two of the major criteria (confirmed by histological evaluation, biochemical testing or imaging) or one major and one supplementary criterion can be diagnosed [2, 5, 7]. Genetic analysis is helpful for the diagnosis of CNC. More than 70% of CNC patients have *PRKAR1A* gene mutations. To date, at least 130 mutations involving 10 exons and adjacent intron sequences have been reported. These mutations (nonsense or missense mutations, short frameshift insertions or deletions and rare large fragment deletions) lead to the loss of functions of the PKA regulatory subunits and the unrestricted activity of the catalytic subunits, resulting in cell proliferation and tumor formation. In addition to the *PRKAR1A* gene, mutations in the *PRKACB* and *PRKACA* genes encoding PKA β and α catalytic subunits, respectively, are also related to the pathogenesis of CNC [5, 7]. Our patient presented with typical features of CNC, including spotty skin pigmentation, thyroid carcinoma and a reported pathogenic mutation of the *PRKAR1A* gene (c.682C > T) in exon 7 [8, 9], which met 2 major criteria and 1 supplemental criterion. Taken together, the diagnosis was clear.

**Table 2:** Diagnostic criteria of CNC

Major criteria
(1) Spotty skin pigmentation with a typical distribution (lips, conjunctiva and inner or outer canthi, vaginal and penile mucosa);
(2) Myxoma (cutaneous and mucosal);
(3) Cardiac myxoma;
(4) Breast myxomatosis or fat-suppressed MRI findings suggestive of this diagnosis;
(5) PPNAD or paradoxical positive response of urinary glucocorticosteroids to dexamethasone administration during Liddle’s test;
(6) Acromegaly due to GH-producing adenoma;
(7) Large cell-calcifying sertoli cell tumors (LCCSCT) or characteristic calcification on testicular ultrasonography, in a young patient;
(8) Thyroid carcinoma or multiple hypoechoic nodules on thyroid ultrasonography;
(9) Psammomatous melanotic schwannoma;
(10) Blue nevus, epithelioid blue nevus (multiple);
(11) Breast ductal adenoma (multiple);
(12) Osteochondromyxoma of bone
**Supplementary criteria**
(1) Affected first-degree relatives;
(2) Inactivating mutation of the *PRKAR1A* gene

Diagnosis of adenomas and tumors requires corresponding histological confirmation

Approximately 20–40% of patients have cardiac myxoma, which is the leading cause of mortality in over 50% of CNC patients [2]. Therefore, clinicians should pay attention to the consultation and physical examination of CNC-related symptoms in patients with cardiac myxoma. Our patient was previously diagnosed with "atrial mucinous adenocarcinoma" due to the postoperative pathology. Given that primary atrial mucinous adenocarcinoma is extremely rare [10], and no cases with CNCs have been reported, we performed immunohistochemistry to confirm the diagnosis. The positive calretinin and vimentin and negative MOC-31 proved that the cardiac myxoma with glandular differentiation was mesenchymal-derived (Fig. 4 C, D, E), so the final revised diagnosis should be "atrial adenomyxoma". A cardiac adenomyxoma is rare, affecting females and the left atrium predominantly. It may be derived from the glandular differentiation of foregut embryonic remnants or myocardial precursor cells with multi-differentiation potential [11]. The clinical features are similar to classic cardiac myxoma but may have recurrence and metastasis [12, 13], emphasizing the necessity of a long-term follow-up observation of this patient.

PPNAD accounts for 45–70% of CNC-related endocrine tumors, leading to adrenocorticotropic hormone (ACTH)-independent Cushing syndrome [7]. The characteristic imaging manifestations are bilateral adrenal pigmented nodules, and the final diagnosis depends on adrenal pathology [7]. This patient had typical features of Cushing syndrome, diabetes, hypertension, and osteoporosis that do not match her age. Cortisol (8:00 am) could not be suppressed by a 1 mg dexamethasone overnight test and a low-dose dexamethasone suppression test, therefore arriving at the diagnosis of Cushing syndrome. However, her ACTH (8:00 am) level was normal, the high-dose dexamethasone inhibition test failed to inhibit below 50% of the baseline level of cortisol (8:00 am), and adrenal CT showed bilateral lesions, which indicated that either Cushing disease or ectopic ACTH/corticotropin releasing hormone (CRH) syndrome could not be excluded. Bilateral inferior petrosal sinus sampling (BIPSS) should be the gold standard for a differential diagnosis. However, the patient refused this invasive test. At the same time, the patient also refused to undergo adrenal surgery, leaving us without pathological findings to confirm the diagnosis of PPNAD. Considering the clear diagnosis of CNC and the presence of PPNAD in almost all CNC patients who underwent autopsy [5], it is speculated that the adrenal glands lesions are PPNAD-related. Previous studies have reported that ACTH may be at a normal or high normal level (more than 20 pg/ml) in PPNAD patients with periodic or irregular hypercortisolism (known as the cyclic Cushing pattern) [14, 15], which also supports our hypothesis. There are very rare case reports indicating that CNC patients might have pituitary ACTH tumors without PPNAD [16], or two types of Cushing syndrome appeared one after another [17], therefore, a long-term follow-up observation is very necessary for our patient. There is currently no specific treatment for the genetic defects of CNC. Surgeries for removing tumors in different locations or drugs such as ketoconazole, mitotane, and osilodrostat are recommended [7, 14]. In addition, the LH, FSH, and E2 levels of this patient were high. A previous study showed that only 20.4% of patients with empty sella syndrome will develop central hypogonadism [18]. Our patient was considered perimenopausal according to the gynecological consultation in her first visit and was postmenopausal 1 year later, which could explain why the changes in her LH, FSH, and E2 levels.

CNC is a rare multiple endocrine neoplasia syndrome. When patients present with clinical features involving multiple endocrine and non-endocrine tumors, especially Cushing syndrome and cardiac myxoma, CNC should be considered. The ability to recognize CNC is crucial for the early diagnosis and prevention of severe complications. Genetic analysis is also recommended in patients with suspected CNC. All patients must be followed up for life.

## Supplementary Information

**Additional file 1:** **Figure S1.** Thyroid fine needle aspiration (FNA) showed a cell group with nuclear crowding, irregularities in the nuclear membrane, intranuclear inclusions and nuclear grooves, which indicated suspicious papillary thyroid carcinoma, Bethesda V.

**Additional file 2:** **Figure S2.** Postoperative thyroid ultrasound and breast ultrasound at follow-up. (A) Postoperative thyroid ultrasound showed a postoperative image of the right thyroid carcinoma. (B) Postoperative thyroid ultrasound showed the left thyroid lobe was negative. (C) Breast ultrasound at follow-up showed multiple hypoechoic nodules in the right breast, cord-like slightly hyperechoic band, maximum size of 19×6 mm, regular in shape, clear in boundary, no blood flow signal, with a BI-RADS classification of 3.

## Data Availability

The raw sequence data reported in this paper will be made available by the corresponding author without undue reservation and have been deposited in the Genome Sequence Archive (Genomics, Proteomics & Bioinformatics 2021) in National Genomics Data Center (Nucleic Acids Res 2022), China National Center for Bioinformation/ Beijing Institute of Genomics, Chinese Academy of Sciences (GSA-Human: HRA003792) that are publicly accessible at https://ngdc.cncb.ac.cn/gsa-human.

## Abbreviations

CNC: Carney complex
PPNAD: Primary pigmented nodular adrenocortical disease
PKA: Protein kinase A
TI-RADS: Thyroid imaging reporting and data system
FNA: Fine needle aspiration
BI-RADS: Breast imaging reporting and data system
DXA: Dual-energy X-ray absorptiometry
ACTH: Adrenocorticotropic hormone
CRH: Corticotropin releasing hormone
HE: Haematoxylin and eosin
BIPSS: Bilateral inferior petrosal sinus sampling
