# Supplementary figures and images for: A case report and literature review of Carney complex with atrial adenomyxoma
Source: BMC Endocr Disord. 2023 Feb 6;23:35. doi: 10.1186/s12902-023-01285-7 (PMC9901083; doi:10.1186/s12902-023-01285-7)

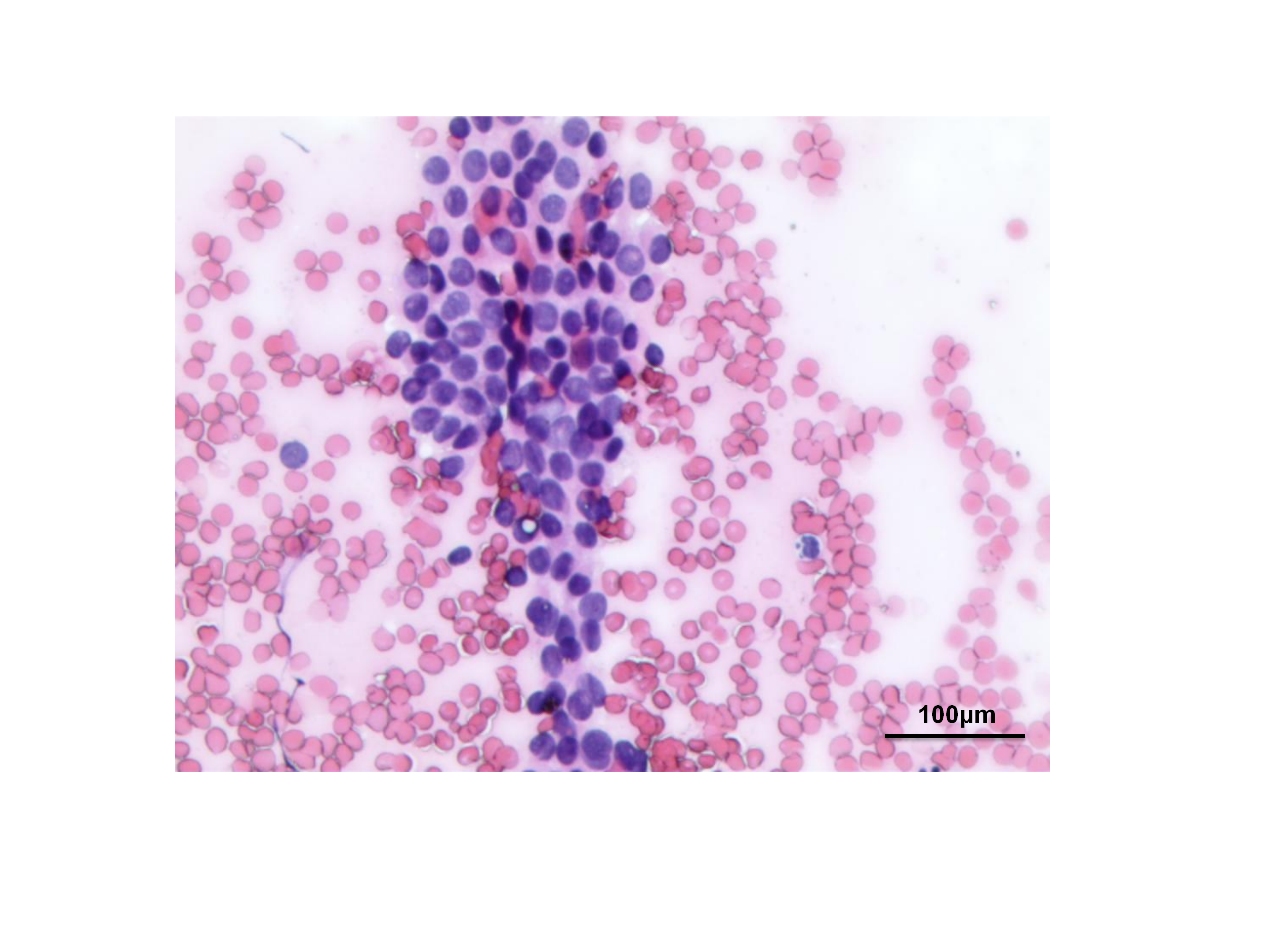

Supplement: Supplementary file 1 — Additional file 1: Figure S1. Thyroid fine needle aspiration (FNA) showed a cell group with nuclear crowding, irregularities in the nuclear membrane, intranuclear inclusions and nuclear grooves, which indicated suspicious papillary thyroid carcinoma, Bethesda V. [file 12902_2023_1285_MOESM1_ESM.tif]

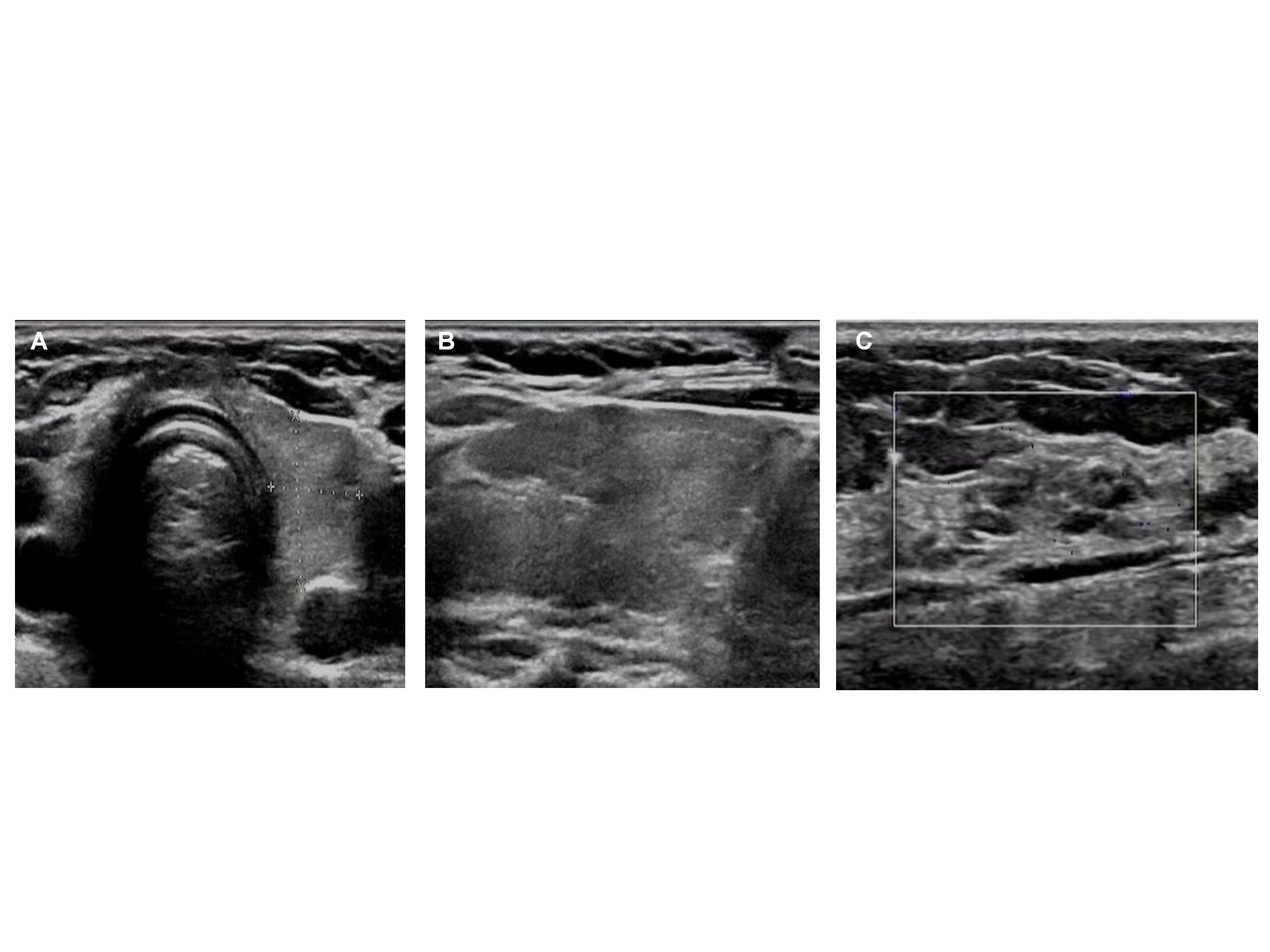

Supplement: Supplementary file 2 — Additional file 2: Figure S2. Postoperative thyroid ultrasound and breast ultrasound at follow-up. (A) Postoperative thyroid ultrasound showed a postoperative image of the right thyroid carcinoma. (B) Postoperative thyroid ultrasound showed the left thyroid lobe was negative. (C) Breast ultrasound at follow-up showed multiple hypoechoic nodules in the right breast, cord-like slightly hyperechoic band, maximum size of 19×6 mm, regular in shape, clear in boundary, no blood flow signal, with a BI-RADS classification of 3. [file 12902_2023_1285_MOESM2_ESM.tif]
